# Pulmonary Consumption—Quackeries of St. John Long

**Published:** 1829-01-01

**Authors:** 


### PULMONARY CONSUMPTION—ST. JOHN LONG.

The mendacious statements which have been set forth in the Literary Gazette and other newspapers respecting Charlatan Long, (who, being unable to succeed as a PAINTER, turned DOCTOR, and is now (or was lately) driving a most lucrative trade,) are too numerous and too greedily swallowed by the public, to admit of sudden refutation. We were going to say that Mr. Long, like most other Charlatans of the day, would make work for the regular practitioners; but we fear that he will leave little for them to do, as far as his own patients are concerned! It is kill or cure with his system of treatment. When once a *really* consumptive patient is *booked* in Harley Street, he cannot too soon pay a visit to the "MEDICAL and CLERICAL LIFE ASSURANCE OFFICE." If the TRUTH should ever come out, the Painter's Lazaretto will be found to resemble, in one respect at least, the cave of Trophonius;—"*nulla vestigia retro*:"—that is, the consultants of the oracle always returned "heels foremost"—a mode of retrogression which is still observed upon certain solemn occasions that need not here be specified.

But we have now to draw the attention of our professional brethren to the principal means employed by this empiric—or, more properly speaking, this Charlatan. Notwithstanding the oath which he imposes on his patients, not to reveal any particulars of their treatment, yet we have had so many opportunities of seeing his deluded disciples, that we can pretty confidently speak as to the means which he employs.

In the first place, the DOCTOR undertakes to cure all consumptive patients, whatever be the extent of disorganization in the lungs, *provided* they have, what he calls, "stamina of constitution." By this state of constitution the learned doctor means, the capability of eating from one to five pounds of animal food daily! If they can do this, without inconvenience, he promises to cure them! If not —he will only try his best. The other measure is a most violent and painful counter-irritation excited on the chest. The precise application used, we have not been able to ascertain—nor is it of any great consequence to be known. We have reason to believe, that it is one of the undiluted, or very slightly diluted, *mineral acids*. The pain is considerable —and the excoriation sometimes frightful. The expressions used by the patients, upon these occasions, are often sufficiently indicative of the agony experienced. We shall give a single specimen. "Oh, Dr. Long! would that I were in Abraham's bosom!" It is in this way that the Charlatan boasts, through the medium of his literary organ, Mr. Jerdan, that he could, at any time, extract a pint of matter from the seat of the complaint—viz. the lungs! The mode of managing his patients, and some very curious phenomena attendant thereon, will probably be best understood by the outline of a case which we had an opportunity of watching.

A gentleman, turned of 30 years of age, came up from an aguish part of the country to London, in the Summer of 1828, labouring, at once, under intermittent fever and symptoms of pulmonary consumption. There was cough, purulent expectoration, morning perspirations, and all the usual symptoms; while a careful examination of the chest shewed a bad state of the right lung, and distinct pectoriloquism about three inches under the right clavicle. There was great difficulty in stopping the paroxysms of ague, as the quinine and arsenic could not well be borne, on account of the disease in the lungs and the irritable state of the bowels. Under these circumstances, we sent him to Hastings, and there the ague hung upon him for some time, and the complication of complaints reduced him, of course, to a state of considerable emaciation.

It was at this period that he was induced to come up from Hastings, and put himself under the care of Mr. Long, who readily undertook the cure. The means were, what we have already mentioned—a most powerful irritant to the surface of the chest—as much animal food as the patient could eat—open air. Now it is a curious fact that, the hope of a cure, the stimulation to the chest, and the recent recovery from a harrassing ague, all combined to produce such an appetite that the patient ate from three to five pounds of animal food every day! In seven weeks of this treatment he gained 30 pounds in weight—his bowels were regular—and his colliquative sweats disappeared! It was now that the patient was induced (and we need not wonder at the circumstance) to sign a paper purporting that he was either cured or on the point of being cured. He had scarcely affixed his signature to the paper when the golden vision began to fade away. A tremendous bowel-complaint came on —the rectum was protruded—Dr. Long was abandoned—and we were once more summoned to the patient. We scarcely recognized him, so much were his features altered, and his face bloated. We directed him to confine himself to bed—applied leeches to the protruded piles, for they could not be returned, and prescribed the usual remedies for the dysentery. We now examined the chest. *The lungs were in precisely the same state as when the patient left us for Hastings. The pectoriloquism was unequivocal—the expectoration was completely purulent.* Here, then, we find that, by a blind empiricism the general system was forced into an unnatural state of plethora and bloated flesh, till a certain point was attained, when Nature could no longer bear the outrageous repletion, and a gorged, or rather inflamed state of the whole internal surface of the bowels, induced a dysentery that ran down the machine with still greater rapidity than that with which it had been wound up. All this time, the excavation in the lungs remained and still remains IN STATU QUO. It is here proper to notice some curious phenomena that occurred during this cannibal system of cure. The first inconvenience which the patient experienced was such a fetor of the perspiration as rendered it very unpleasant to be in the same room with him. This inconvenience, he bore patiently, under the hope of cure; but the tongue became like a beef-steak—and then the dysentery soon dissipated the fond illusion of a cure forced in the above manner! Another remarkable fact was noticed, not only in this case, but in one or two other of Mr. Long's patients. It was the keen appetite which succeeded the torturing applications to the chest. More than one of Mr. Long's patients declared to us that it would have been quite impossible for them to eat the quantity of food they did, had it not been for the applications to the surface of the chest. This is a fact from which some important practical indications may be drawn. That temerity and ignorance which led Mr. Long to look upon a voracious appetite and increase of flesh as the main desiderata, (though they must often be destructive to the lungs ultimately,) may have struck out a *means* of procuring this appetite which cautious science might never have discovered. We recommend, therefore, that our brethren, especially those in hospital practice, should try the effects of the mineral acids applied to the thoracic parietes, where counter-irritation is deemed necessary, and to watch the effects on the functions of the stomach.

A third phenomenon is worthy of record. During the time that these irritating applications were making to the chest, and that animal food was taken every two or three hours, even in the night, the bowels became firm—the motions large and formed. This, of course, had an end. At the expiration of seven weeks, the bowels gave way, and the dysentery endangered life.

For some years past we have advocated the propriety of giving animal food and tonics, (whenever they can be borne,) when there is actual breach of structure in the lungs, with wasting expectoration and night-sweats. The whole of Mr. Long's success has hinged upon this treatment; though, from his ignorance of the pathology of the disease, he has employed the means indiscriminately—and consequently must have been more frequently wrong than right. But, as we said before, the scientific practitioner may take hints from these dangerous Charlatanries, which may prove of great use in the healing art.

P.S. The following extract of a letter from a gentleman at Penzance (a quondam patient of Long's) to his friend near London, communicated to us lately, will prove amusing, if not instructive.

"I dare say you have heard much of the humbug quack, LONG, since I left London. Another of his patients died about a fortnight before I quitted him. He always came in a gig. He had been dead a week before the unfeeling wretch took the trouble to inquire about him, although he knew how weak he was the last time he came—nor would he have then inquired, but that two or three of us told him of his unfeeling conduct. This man died worth *twenty-six thousand pounds*, and with no relation to leave it to!

"How is the Rev. Mr. N———n? Is he still under Long? Mr. Long's patients were dropping off very fast when I

left. Mr. H——— was going to the West Indies. Mr. N——— had left. Capt. G——— and another about leaving. Mr. R———y had left.

"I am quite delighted with this place; (Penzance) it is beautifully situated—the air is mild and muggy. I have taken a warm-bath almost every day since I came here. The situation is well suited for an invalid. I am already much better. My cough has almost entirely left me. There are few places to equal Penzance for the salubrity of the air, and the beauty of the scenery." J. C.

We can assure our professional brethren, that the bubble of charlatan Long's reputation is already burst, and we venture to predict, that in one short year, the Lazaretto in Harley Street will be as silent as the ruins of Palmyra.

